# The development of the Polish version of the Compassionate Engagement and Action Scales

**DOI:** 10.1371/journal.pone.0323687

**Published:** 2025-05-15

**Authors:** Mariusz Zięba, Julia E. Wahl, Mateusz P. Zatorski, Paul Gilbert

**Affiliations:** 1 Institute of Psychology, SWPS University, Poznań, Poland; 2 Institute of Human Biology and Evolution, Faculty of Biology, Adam Mickiewicz University, Poznań, Poland; 3 College of Health and Social Care Research Centre, University of Derby, Derby, United Kingdom; Universiti Sains Malaysia - Kampus Kesihatan, MALAYSIA

## Abstract

Compassion has been a subject of extensive scientific research for over two decades. There is clear evidence that our capacity for compassion evolved out of care motivation. Like all motivations it is operated via stimulus response algorithms. For compassion motivation stimulus sensitivity focuses on the processing of indicators of suffering, distress and need, called engagement. The response functions switch attention and processing to what is likely to be helpful in alleviating suffering, distress and need, called action. The Compassion Engagement and Action Scales (CEAS) were developed to measure the S-R algorithm of compassion. Because compassion, like other psychological phenomena can operate interpersonally and intrapersonally, there are three scales that give separate assessments for directing compassion to 1. the self, 2. others and 3. responsiveness to compassion from others. They have been used in many international studies and there is now substantial evidence. The research aimed to validate the CEAS within a Polish population. The three cross-sectional studies involved a total of 1,219 participants from Poland. Confirmatory factor analysis conducted on two separate samples indicates that bifactor models provide the best fit for both the Compassion for Others scale and the Compassion from Others scale. In the first, the model includes a general compassion for others factor alongside specific factors for engagement and actions. Similarly, the second features a general compassion from others factor with the same specific factors. This means that being sensitive to suffering and taking action represent specific components of compassion. However, the bifactor model for Compassion for Self requires further refinement due to lower fit indices and the need for item adjustments. The study results generally support the reliability and validity of the CEAS-PL across diverse samples, aligning with findings from previous studies on the original tool and its language adaptations. Notably, tests of validity—including correlations with emotion regulation, well-being, and attachment styles—highlighted distinct patterns for the three flows of compassion, underscoring their conceptual independence. The CEAS-PL shows promise as a valuable tool for psychological research and practice, especially in the areas of pro-social behaviour and helping people with mental health problems, facilitating the assessment of compassion across different orientations. It may support practitioners in identifying individual competencies and tailoring interventions to enhance compassion-related competencies to address particular difficulties.

## Introduction

The nature and value of compassion for personal welfare and moral behaviour have been recognised for thousands of years [1]. However, it is only comparatively recently that compassion has been subjected to scientific study [2–4]. Although there are some differences in how compassion is defined [5,6], there is general agreement, from the contemplative traditions [1,7] and their scientific study that compassion is motivation that emerges from caring behaviour [8,9]. All motivations are underpinned by attention to certain stimuli that triggers corresponding responses. For example, the motive to stay safe means that organisms are attentive to threat cues which then trigger defensive behaviours. If they are motivated by sexual activity or feeding, organisms will be *attentive* to particular stimuli indicating that potential, and then physical and behaviourally accordingly. The evolution of caring led to the development of the ability to be attentive to the needs of others, particularly those in distress, and to respond appropriately. In attachment relationships, for example, a parent, attentive to their infant’s needs or distress, undergoes physiological change in response to these signals, and responds according to the nature of the distress or need. For example, this may include feeding, temperature regulation, physical comforting/soothing, or rescuing. Compassion evolved from caring behaviour, but differs from it, because it is also shaped by recently evolved cognitive and empathic abilities [10]. Conceptualising compassion as a basic motive (not an affect) with both stimulus detection (e.g., of. distress or need) and response functions means it can be defined a ‘sensitivity to suffering in self and others with a commitment to try to alleviate and prevent it’ [2]. It is important to distinguish the stimulus detection from the response functions since they are linked to different physiological processes and different skills [5,10,11]. For example, Di Bello et al [12] showed that there were different physiological responses to viewing a distressing scenario with sensitivity, compared to viewing the same scenario while thinking of what would be helpful (response functions). Emotions associated with compassion are context-dependent. For example, the emotions of a firefighter risking their life to save somebody will be very different to those of a counsellor working with a patient dying of cancer, or a compassionate parent setting boundaries for their child.

To explore the properties and effects of compassion requires appropriate assessment of compassion. This in turn depends upon the conceptualization and definition of compassion. For example, an approach labelled mindful self-compassion takes a different view to rooted in evolved motivational systems and focuses on three dimensions of expressions of self-compassion regarded as bipolar 1. kindness versus self-criticism. 2 common humanity versus sense of isolation and aloneness. 3 mindfulness versus absorption/rumination [13]. This approach generated the self-compassion scale to measure these dimensions [14]. Recently Pommier et al., [15] developed this model to measure compassion *to others*. Another approach has explored compassion in terms of different components and skills such as distress tolerance and empathy derived from a systematic review of the compassion literature [16] which generated the Sussex-Oxford compassion scales [17].

Using the evolved motivation approach (5) enable exploration of the two core qualities of compassion 1. ability to be sensitive, attentive (rather than avoidant) to suffering distress and need, and 2. working out appropriate responses. These, in turn can be broken down into specific skills, such as those related to sensitivity and engagement with suffering: mindfulness, empathy for distress, and distress tolerance. For the response function there are skills such as: attention to and using knowledge about what it is like to be helpful, empathy for the helpful, distress tolerance generated by the actions required (for example, a firefighter has to tolerate anxiety when entering a house, whereas a counsellor has to tolerate working with a dying client). This approach generated a measure called the Compassion Engagement and Action Scales [18] to measure these two quite different dimensions [12].

Compassion can also be conceptualised as a flow of internal and social interactions: there is the compassion we feel for others, the compassion we receive from others, and the compassion we hold for ourselves. Hence, the two dimensions of compassion can be explored in relationship to flow. For example, we can be sensitive to distress of others and work on how to address it, or choose not to. Poulin [19] indicated that people often know what to do to be compassionate, but they do not do it. We might know how to stand up against social injustice but be too fearful. Gilbert et al. [18] found that sensitivity to distress on its own, without taking any action, actually increased vulnerability to depression and anxiety. Therefore, being sensitive and empathetic may not be enough to be compassionate, especially if it leads to feeling overwhelmed or to a downward spiral [1].

The conceptualisation and measurement of compassion are crucial if we are to explore the impact of compassion training and its variables. The CEAS has now been used in many international studies and populations [20].

Previous adaptations of CEAS included Japanese [21], Turkish [22], Dutch [23], and Slovak [24] language versions. As part of the research project *Compassion, social connectedness and trauma resilience during the COVID-19 pandemic: A multi-national study* [20,25], the measure was also translated into 21 languages. A study re-verifying the psychometric quality of the CEAS on a sample of the UK population was also conducted [26].

To date, there has been no study of a translated CEAS in Polish. Additionally, we aimed to explore how this translation interacts with relationship processes. This is important because translating concepts and definitions of psychological processes across languages does not always fully capture their intended meaning. For example, in Polish, the term compassion (Pol. *współczucie*) refers to “emotional solidarity with the sufferer” [27], which can be translated into psychological terms such as empathic concern or sympathy. It is important to note that the word “solidarity” itself connotes something shared, cooperative, and interdependent. In Polish, the term may also imply not being on an “equal footing” with the person experiencing distress. A key aspect of the Polish word “współczucie” is that the prefix “współ” means joined or common, suggesting that “współczucie” could be translated as “common-passion” [28]. Martha Nussbaum [29] argues that compassion for another requires a belief in common humanity, which aligns with the definition of the word *compassion* in Polish–which also includes the concept of solidarity.

In other cultural realms there may be other connotations that play a role. For instance in Tibetan, the word *nyingje* is never singular which interestingly corresponds with the idea of compassion always being all-directional and relational (i.e., self and others). This shows that different linguistic spheres may emphasize different aspects of and dimensions to compassion that are not present in other languages. Thus, this can influence individuals’ understanding of what compassion entails and how it should be exhibited [28]. Moreover, it reveals one’s understanding of their hypocognitive experiences as lack of conceptual knowledge confines what people perceive, are aware of, and how they retain that information [30].

The present study aimed to adapt the CEAS for the Polish language, verifying the validity and reliability of the scale, with its three distinctive scales, referring to the three orientations of compassion, namely: compassion for oneself (self-compassion), compassion for others, and compassion from others. Each scale also measure the two processes (or psychologies) of compassion: engagement with compassion to suffering and taking action for the cessation of suffering.

## Methods

The aim of the presented study was to examine the psychometric properties of the Polish version of the Compassionate Engagement and Action Scales (CEAS-PL). We conducted a cross-sectional, online study using Qualtrics (https://www.qualtrics.com/). Participant recruitment for the study took place between 24/08/2021 and 31/10/2021. Informed consent and detailed information about the study were provided on the introductory page of the online questionnaire. To ensure confidentiality and anonymity, written consent was not required. Participants were informed that by proceeding with the questionnaire, they were agreeing to participate in the study, and they were asked to respond only if they fully agreed to take part. The study procedure was approved by the university's research ethics committee (no. 2021-80-11), and data were collected in accordance with the 1964 Helsinki Declaration.

### Data analysis

Data analysis was conducted using IBM SPSS Statistics for Windows, Version 29.0 [31], and JASP, Version 0.19 [32], with the application of lavaan [33].

#### Factor structure and reliability.

To conduct a preliminary exploration of the data structure, we performed Exploratory Factor Analyses (EFA) using the maximum likelihood method for each of the three CEAS-PL scales. We removed items with factor loadings lower than 0.40, as they indicated inadequate representation of the underlying factor [34].

To evaluate the factorial structure of the CEAS-PL, we compared several models using Confirmatory Factor Analysis (CFA). Model fit was assessed using the normed Chi-Square (χ²/df), comparative fit index (CFI), Tucker-Lewis Index (TLI), root-mean-square error of approximation (RMSEA), and standardized root mean square residual (SRMR). The normed Chi-Square (χ²/df) value below 3 indicates a good fit [35], and values below 5 are considered acceptable [36]. Good model fit is indicated by indices of RMSEA < .07 [37], SRMR < .08 [35], and CFI and TLI > .90 [35]. A combination of absolute fit indexes (e.g., χ²/df, RMSEA, SRMR) and incremental fit indexes (e.g., CFI, TLI) is recommended to assess model fit, providing a comprehensive evaluation of the model's performance [38].

When comparing models, we used the ΔCFI index, with values lower than.01 indicating no statistically significant difference in model fit [39].

Internal consistency was assessed by calculating Cronbach’s alpha, with values above.70 considered acceptable and values above.90 considered excellent [40]. Test-retest reliability was evaluated using a bivariate correlation between the initial measurement and a repeat administration 15 months later. For measurements taken after such a long interval, reliability scores above.70 are considered acceptable [40].

#### Construct validity.

We tested the validity of the CEAS-PL through Pearson’s correlation coefficients to evaluate convergent and discriminant validity. Effect sizes were interpreted based on Cohens’s [41] indicators: correlations of ≧.10 were considered small, ≧ .30 medium, and ≧.50 large.

Based on the literature, including validation studies of the original CEAS [18] and other language versions (21–24,26), we hypothesized that compassion for self would have a large positive correlation with the Self-Compassion Scale [14] and a moderate positive correlation with the Compassion Scale [15] measuring compassion for others. For the other two CEAS-PL scales, we expected high positive correlations with the Compassion Scale and empathic concern and moderate correlations with the Self-Compassion Scale. We also anticipated that all CEAS-PL scales would be moderately positively correlated with life satisfaction, well-being, emotion regulation, close attachment orientation, extraversion, and openness to experience, and moderately negatively correlated with depression, anxiety, stress, and neuroticism. Weak correlations or no relationship were expected between the three CEAS-PL scales and conscientiousness.

### Participants

The participants were adults who volunteered to take part in the study, and the exclusion criterions were current participation in psychiatric treatment, psychotherapy, or compassion training. Three different populations were recruited for this study:

Sample 1 included 593 psychology students from 5 different campuses of SWPS University in Poland. The participants ranged in age from 18 to 55 years (*M* = 26.33, *SD* = 8.18) and consisted of 517 women, 73 men, and 3 individuals who identified as another gender.

Sample 2 included 238 adults living in various parts of Poland, aged 18–74 years (*M* = 36.14, *SD* = 15.77). This sample consisted of 167 women, 68 men, and 3 individuals who identified as another gender. Among the participants, 92 were full-time employees, 32 worked part-time or on a contract, 50 were students, 12 owned businesses, 33 were retired or on disability, 15 were unemployed, and 34 were homemakers or on parental leave.

Sample 3 consisted of 378 adults living in various parts of Poland. Ages ranged from 18 to 67 years (*M* = 26.65, *SD* = 7.94). Among the participants, 210 were female, 165 were male, and 3 identified as another gender. Education levels included 16 with primary and vocational education, 98 with secondary education, 131 pursuing higher education, 129 completed higher education, and the remainder declared “other” forms of education.

### Measurement

#### Compassionate Engagement and Action Scales (CEAS-PL).

Before conducting the validation study of the Polish version of the Compassionate Engagement and Action Scales (CEAS-PL), permission was obtained from the original authors of the tool [https://www.compassionatemind.co.uk/] to carry out an adaptation. The first stage of cultural validation [42,43] involved forward translation. The original questionnaire was translated from English to Polish by three independent individuals whose native language is Polish [44]. The translators included an English teacher who was also a final-year psychology student, and a scholar with PhD in psychology. The next step was reconciliation. The validation team—comprising MZ, JW, and MPZ—analysed the resulting translations to ensure they aligned with the theoretical assumptions underlying the concept of compassion. This process resulted in a single version of the questionnaire, which was then back-translated in the next step. The validation team re-examined the original version, the standardized translated version, and the back-translated version. Due to linguistic uncertainties in three questions on the Compassion for Others scale, two independent native English speakers were asked to retranslate these items from Polish back into English. After this additional retranslation, the items were compared again, and it was concluded that the Polish version was equivalent to the original English version.

The final version of the CEAS tool was selected by the validation team, based on the translation that was both theoretically accurate and linguistically closest to the original. This final version of the questionnaire was then subjected to cognitive debriefing. Several individuals with varying levels and fields of education (including higher and secondary education, as well as humanities and technical studies) assessed the clarity of the instructions and questions. Feedback indicated that, while the questionnaire was not easy (e.g., it required time to provide thoughtful, reflective answers), the instructions and questions were understandable.

As in the original scale [18], the CEAS-PL consists of three subscales, each corresponding to a different form of compassion flow: toward oneself, toward others, and from others, in accordance with the underlying theory. Each subscale is divided into two sections. The first section contains 8 statements related to motivation and commitment in coping with discomfort and tension, while the second section includes 5 statements focused on the use of compassion in dealing with emotions, thoughts, and situations that cause discomfort and tension. For example, a question representing self-compassion in terms of motivation and commitment might be: “I accept, do not criticise and do not judge my tension and discomfort*”*. An example from the same subscale, but representing the use of compassion in coping, could be: “I direct my attention to what might be helpful to me”. Respondents select their answers on a scale from 1 to 10, indicating how often they behave in the manner specified in the question ranging from *never* to *always*).

The Supplementary Materials include the CEAS-PL questionnaire (File 1), as well as the wording of each item in both the original and Polish versions, along with the item codes used in the present article (File 2).

#### Self-Compassion Scale (SCS).

The SCS is based on Neff’s [45] self-compassion model. Its development involved qualitative research (focus groups) followed by studies verifying the scale’s validity and reliability [14]. The original tool, as well as the Polish adaptation used in this study [46], consists of six subscales: Kindness to Self, Judging Self, Community of Experience, Isolation, Mindfulness, and Over-identification. In our study, we used three positively oriented subscales: Kindness to Self, Community of Experience, and Mindfulness. Each subscale includes 4 items rated on a 5-point scale from *almost never* (1) to *almost always* (5).

#### Compassion Scale (CS-R).

The Compassion Scale measures compassion for others, operationalized as experiencing kindness, a sense of common humanity, mindfulness, and reduced indifference toward others’ suffering [15]. Although initially conceptualized to be similar to self-compassion [13], compassion for others differs, especially in terms of the lack of compassion, which manifests as perceptual, emotional, and cognitive indifference [15]. This is especially relevant to the dimension reflecting a lack of compassion toward others, which involves inattentiveness, emotional disengagement, and cognitive distancing. The tool consists of four subscales: Kindness to Others, Community of Experience, Mindfulness, and Indifference. Each subscale contains 4 items rated on a 5-point scale from *almost never* (1) to *almost always* (5), with the Indifference subscale requiring reverse scoring. The Polish adaptation [47] was used in this study.

#### Empathic Sensitiveness Scale (ESS).

The ESS is a questionnaire developed by Polish researchers [48], based on Davis’s [49] concept of empathy. Davis defines empathy as a cognitive, affective, and behavioural reaction of an observer to another person. He created the IRI (Interpersonal Reactivity Index) to measure these reactions [50]. With Davis’s permission, the Polish authors paraphrased the IRI, retaining 17 original questions and adding 11 new ones. The ESS includes three subscales: Emotional Concern, Perspective Taking, and Personal Distress, but excludes the Fantasy scale from the original tool, citing Davis’s own doubts about its validity. Responses are measured on a 5-point Likert scale, ranging from *strongly disagree* (1) to *strongly agree* (5). The EES demonstrates satisfactory psychometric properties, with validity assessed in relation to the Personality Questionnaire 16 PF-5 and the State and Trait Anxiety Inventory (STAI).

#### Revised Adult Attachment Scale (RASS).

The RASS is an 18-item scale measuring attachment styles based on the Bartholomew and Horowitz [51] model. Authored by Collins [52], it classifies individuals into one of four adult attachment patterns: secure, preoccupied, distancing, and anxious. The style is determined by summing scores from three subscales: Close, Depend, and Anxiety, each with six statements rated on a 5-point scale from *not at all typical for me* (1) to *very typical for me* (5). The study used the Polish adaptation by Adamczyk & Pilarska [53], which demonstrates satisfactory psychometric properties similar to the original tool.

#### Emotion Regulation Questionnaire (ERQ).

The ERQ is a 10-item scale based on Gross’s [54] processual model of emotion regulation. According to this model, emotion regulation encompasses both unconscious and conscious strategies that individuals use to enhance, sustain, or diminish emotional reactions [55]. In both the original tool [56] and the Polish adaptation by Kobylińska [57,58] used in the study, participants respond to statements identifying their forms of emotion regulation. Responses are rated on a 7-point scale from *strongly disagree* (1) to *strongly agree* (7).

#### Psychological Well-Being Scales (PWBS).

The theoretical basis of the PWBS is Ryff’s [59] concept of well-being. Ryff’s theory and the questionnaire itself distinguish six dimensions of well-being: self-acceptance, personal growth, purpose in life, autonomy, environmental mastery, and positive relations with others. These dimensions constitute the subscales of the questionnaire, which comes in two versions: 84 items and 18 items. Respondents rate statements on a 6-point scale from *strongly disagree* (1) to *strongly agree* (6) reflecting how they think about themselves and their lives. The study used the Polish 18-item adaptation of the scale [60].

#### Satisfaction with Life Scale (SWLS).

The SWLS was developed by Diener et al. [61] to measure an individual's global cognitive judgments of their satisfaction with life. The scale consists of five items, each rated on a 7-point Likert scale ranging from *strongly disagree* (1) to *strongly agree* (7). It is widely used in psychological research due to its simplicity, reliability, and validity, making it a valuable tool for assessing subjective well-being [62]. We used the Polish adaptation of the scale by Jankowski [63].

#### Depression Anxiety Stress Scales (DASS-21).

The DASS-21 [64] is a brief self-report instrument designed to measure the negative emotional states of depression (feelings of dysphoria, hopelessness, and lack of interest), anxiety (autonomic arousal and situational anxiety), and stress (valuates difficulty relaxing and nervous arousal). The scale consists of 21 items, with seven items for each subscale. Participants rate their experiences over the past week on a 4-point Likert scale, from *did not apply to me at all* (0) to *applied to me very much or most of the time* (3). The DASS-21 is widely used in clinical and research settings due to its strong psychometric properties, providing a reliable and valid assessment of an individual’s emotional state. The study used the Polish adaptation of the questionnaire by Makara-Studzińska and colleagues [65].

#### Ten Items Personality Inventory.

The TIPI is a brief 10-item scale designed to measure personality traits within the Big Five framework [66,67]. The questionnaire comprises ten pairs of adjectives, with two pairs for each trait (extraversion, agreeableness, conscientiousness, emotional stability, openness to experience). Each pair represents opposite ends of a trait dimension (e.g., extraversion vs. introversion). Respondents rate how accurately the adjectives describe them on a 7-point scale from *strongly disagree* (1) to *strongly agree* (7). Scores for each trait are averaged from two items, one of which is reverse-coded. The study used the Polish TIPI adaptation by Łaguna et al. [68]. The authors report no significant reliability differences compared to the original [69] and high validity, with strong correlations to the NEO-FFI [66].

## Results

### Preliminary analyses

We assessed the assumptions for Exploratory Factor Analyses (EFA) and confirmed that the Kaiser–Meyer–Olkin (KMO) measure, Bartlett’s test of sphericity, and determinant values indicated that these assumptions were met for all three scales: Compassion to Self (CtoS), Compassion to Others (CtoO), and Compassion from Others (CfO).

For the CtoS scale, the initial EFA suggested a three-factor solution, but the structure was difficult to interpret due to 3 items loading on multiple factors. We then performed EFA assuming a single-factor solution. Items CtoS_E02, CtoS_E04, and CtoS_E05 had loadings below.40. After excluding these items, the factor loadings ranged from.44 to.90, explaining 54.4% of the variance.

For the CtoO scale, the initial EFA suggested a two-factor solution, with the second factor including only item CfS_E05 and an eigenvalue slightly above 1 (1.05). We then performed EFA assuming a single-factor solution. Item CfS_E05 had a loading below.40. After excluding this item, the factor loadings ranged from.45 to.85, with the factor explaining 54.2% of the variance.

For the CfO scale, a single-factor solution emerged with eigenvalues >1. The factor loadings ranged from.57 to.90, explaining 64.4% of the variance.

### Confirmatory Factor Analysis (CFA)

Before conducting the confirmatory factor analysis (CFA), we assessed the assumptions for using the maximum likelihood (ML) estimation method. Specifically, we tested for multivariate normality and found no significant violations.

In the first step, we conducted a three-factor model (Model I) with ten items relating to CtoS (factor 1), ten items relating to CtoO (factor 2), and ten items relating to CfO (factor 3). The three-factor CFA with all 30 items produced a poor to fair model fit (see Table 1). Parameter estimation of Model I identified four items with factor loadings below.40:

**Table 1:** Indices of fit for two three-factor models.

	χ2/df	RMSEA (90% CI)	SRMR	TLI	CFI	Model comparison	Δ CFI
Model I (three-factor, 30 items)	4.05	.072 (.068 -.075)	.062	.882	.891		
Model II (three-factor, 26 items)	3.71	.068 (.063 -.072)	.044	.917	.925	Model I vs Model II	.34

χ²/df = Chi-square divided by degrees of freedom; RMSEA = Root Mean Square Error of Approximation; SRMR = Standardized Root Mean Square Residual; TLI = Tucker-Lewis Index; CFI = Comparative Fit Index; Δ CFI = Change in Comparative Fit Index. Model I includes all 30 items. Model II excludes items CtoS_E02, CtoS_E04, CtoS_E05, and CtoO_E05, due to low factor loadings.

„Zauważam i jestem wrażliwy/a na moje uczucia napięcia i dyskomfortu gdy się pojawiają/ I notice, and am sensitive to my distressed feelings when they arise in me” (CtoS_E02), “Czuję się poruszony emocjonalnie w stresujących sytuacjach/ I am emotionally moved by my distressed feelings or situations” (CtoS_E04), “Toleruję różnorodne uczucia, które składają się na moje napięcie i dyskomfort (dystres)/ I tolerate the various feelings that are part of my distress” (CtoS_E05), and “Znoszę różnorodne uczucia, które składają się na napięcie i dyskomfort (dystres) u innych ludzi/ I tolerate the various feelings that are part of other people’s distress” (CtoO_E05). These are the same items that had factor loadings below.40 in the previously presented EFA analyses, indicating inadequate representation of the underlying factor. Removal of these four low-loading items resulted in improved fit, with RMSEA below.08 and better SRMR. TLI and CFI values are now above.90, indicating a good fit. The ΔCFI of 0.34 signifies a significant improvement over Model I.

A visual representation of Model II is provided in the Supplementary Material (S1 Fig).

Standardised coefficients for the CtoS factor range from.44 up to.90; for the CtoO factor range from.45 up to.85, and for CfO factor range from.44 up to.90.

**Fig 1:**
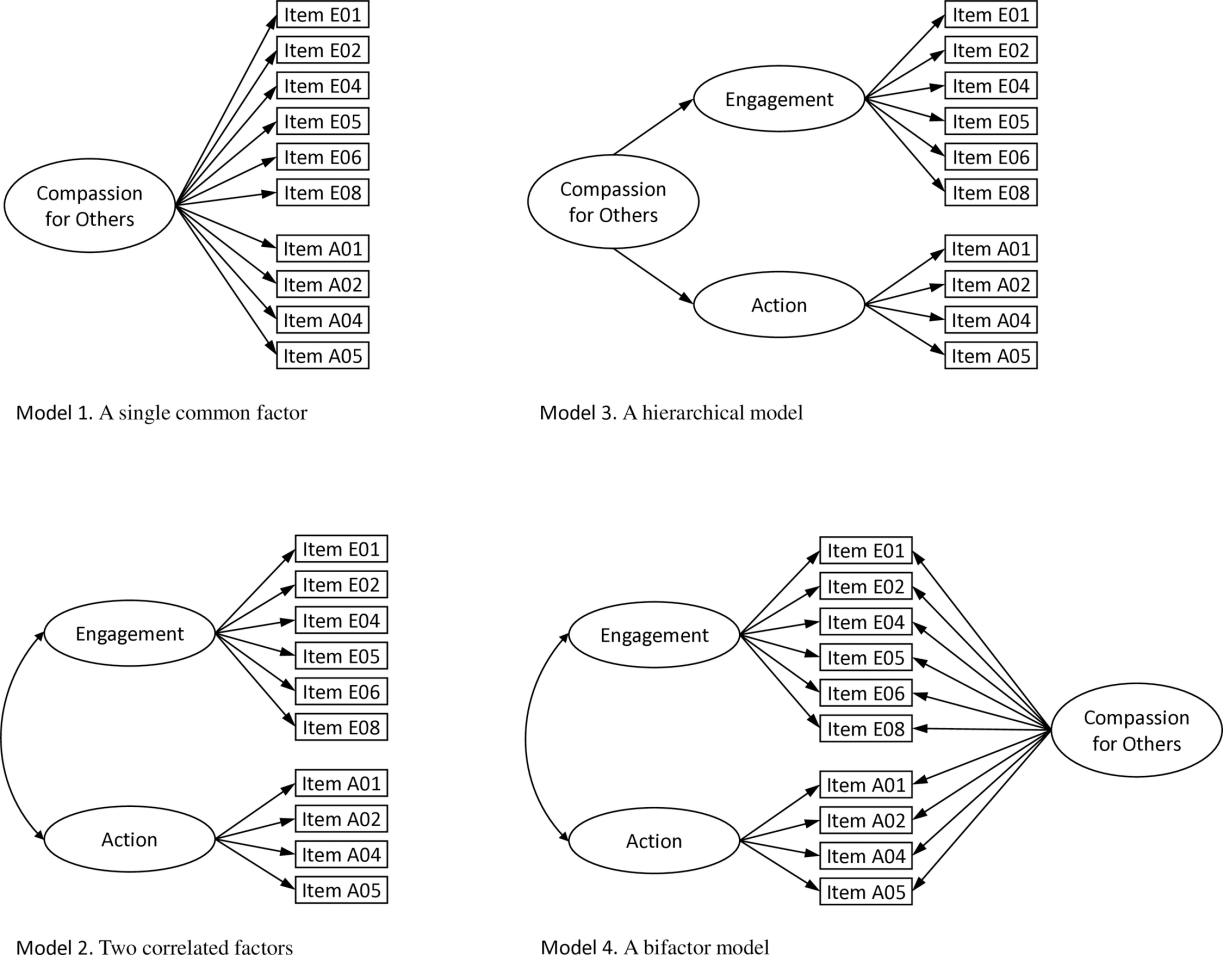
The alternative measurement models for the Compassion for Others scale.

The original version of the CEAS, according to Gilbert et al.‘s [18] concept, was constructed with the assumption that each of the three components (“flows”) of compassion includes six items related to the competencies of compassionate engagement and four items related to the competencies of compassionate action. Based on this structure, we conducted a series of CFAs separately for each of the three scales. For each scale, we compared four alternative models: (1) a single common factor, (2) two correlated factors: engagement and action, (3) a hierarchical model with one second-order factor (compassion) consisting of two first-order factors (engagement and action), and (4) bifactor models assuming the existence of three factors: compassion (comprising all items in the given scale), and two correlated factors, engagement and action, each encompassing three observed variables. The alternative models for Compassion for Others are presented in Fig 1, while the corresponding alternative models for Compassion for Self and Compassion from Others are provided in the Supplementary Materials as Figs S2 and S3, respectively.

In the CFAs analyses, we examined the fit of each model to data from Sample 1 (students, *N* = 593) and Sample 2 (general population, *N* = 238). Table 2 presents the model fit indices for each of the four factor models across the three scales (Compassion to Self, Compassion to Others, Compassion from Others) based on the data from Sample 1, while Table 3 shows the results of analogous analyses for the data from Sample 2.

**Table 2:** Indices of fit for all models of the CEAS-PL structure (sample 1).

	χ2/df	RMSEA (90% CI)	SRMR	TLI	CFI	Model comparison	Δ CFI
Compassion to Self							
Model CtoS_1 – single factor	6.52	.096 (.078 -.116)	.037	.949	.966		
Model CtoS_2 – two correlated factors	6.99	.101 (.082 -.120)	.036	.945	.966	CtoS_1 vs CtoS_4	.026
Model CtoS_3 – hierarchical	8.24	.105 (.086 -.125)	.036	.939	.965	CtoS_2 vs CtoS_4	.026
Model CtoS_4 – bifactor	5.41	.086 (.053 -.123)	.014	.959	.992	CtoS_3 vs CtoS_4	.027
Compassion to Others							
Model CtoO_1 – single factor	9.71	.121 (.108 -.135)	.041	.900	.925		
Model CtoO_2 – two correlated factors	7.55	.105 (.092 -.119)	.037	.925	.946	CtoO_1 vs CtoO_4	.061
Model CtoO_3 – hierarchical	7.86	.108 (.094 -.122)	.037	.921	.945	CtoO_2 vs CtoO_4	.040
Model CtoO_4 – bifactor	3.99	.071 (.053 -.090)	.022	.966	.986	CtoO_3 vs CtoO_4	.041
Compassion from Others							
Model CfO_1 – single factor	17.69	.168 (.157 -.179)	.166	.848	.878		
Model CfO_2 – two correlated factors	4.56	.078 (.065 -.090)	.026	.968	.975	CfO_1 vs CfO_4	.110
Model CfO_3 – hierarchical	4.70	.079 (.067 -.092)	.026	.966	.975	CfO_2 vs CfO_4	.013
Model CfO_4 – bifactor	3.60	.066 (.051 -.082)	.015	.976	.988	CfO_3 vs CfO_4	.013

χ²/df = Chi-square divided by degrees of freedom; RMSEA = Root Mean Square Error of Approximation; SRMR = Standardized Root Mean Square Residual; TLI = Tucker-Lewis Index; CFI = Comparative Fit Index; Δ CFI = Change in Comparative Fit Index.

**Table 3:** Indices of Fit for All Models of the CEAS-PL Structure (Sample 2).

	χ2/df	RMSEA (90% CI)	SRMR	TLI	CFI	Model comparison	Δ CFI
Compassion to Self							
Model CtoS_1 – single factor	1.39	.032 (.000 -.063)	.022	.993	.996		
Model CtoS_2 – two correlated factors	1.44	.034 (.000 -.066)	.022	.993	.995	CtoS_1 vs CtoS_2	.000
Model CtoS_3 – hierarchical	1.56	.039 (.000 -.071)	.022	.991	.996	CtoS_2 vs CtoS_3	.001
Model CtoS_4 – bifactor	N.A.						
Compassion to Others							
Model CtoO_1 – single factor	7.07	.127 (.110 -.144)	.038	.907	.930		
Model CtoO_2 – two correlated factors	5.60	.110 (.093 -.128)	.035	.930	.949	CtoO_1 vs CtoO_4	.051
Model CtoO_3 – hierarchical	5.82	.113 (.096 -.131)	.035	.926	.949	CtoO_2 vs CtoO_4	.032
Model CtoO_4 – bifactor	4.03	.089 (.067 -.114)	.026	.954	.981	CtoO_3 vs CtoO_4	.032
Compassion from Others							
Model CfO_1 – single factor	11.88	.170 (.156 -.184)	.152	.825	.860		
Model CfO_2 – two correlated factors	5.50	.109 (.094 -.125)	.039	.928	.945	CfO_1 vs CfO_4	.129
Model CfO_3 – hierarchical	5.67	.111 (.096 -.127)	.039	.925	.945	CfO_2 vs CfO_4	.044
Model CfO_4 – bifactor	2.43	.062 (.041 -.083)	.016	.977	.989	CfO_3 vs CfO_4	.044

χ²/df = Chi-square divided by degrees of freedom; RMSEA = Root Mean Square Error of Approximation; SRMR = Standardized Root Mean Square Residual; TLI = Tucker-Lewis Index; CFI = Comparative Fit Index; Δ CFI = Change in Comparative Fit Index.

### Reliability

The internal consistency of the CEAS-PL scales was assessed using Cronbach’s α, with the results presented in Table 4.

**Table 4:** Reliability indices for all scales of the CEAS-PL.

	Study 1 (*N* = 593)	Study 2 (*N* = 238)	Study 3 (*N* = 378)
Compassion to Self	,87	,88	,87
Compassion to Self—Engagement	,58	,65	,56
Compassion to Self—Actions	,90	,91	,89
Compassion to Others	,90	,92	,91
Compassion to Others—Engagement	,80	,84	,80
Compassion to Others—Actions	,89	,91	,92
Compassion from Others	,94	,92	,93
Compassion from Others—Engagement	,89	,89	,86
Compassion from Others—Actions	,94	,84	,92

The test-retest reliability of the CEAS-PL scales was examined in a subsample of the study participants from Sample 1 (*N* = 30) over a 15-month period. The one-tailed Pearson correlation coefficients between the first and second measurements are.62 for Compassion to Self,.64 for Compassion to Others, and.57 for Compassion from Others, all significant at *p* < .001.

The results of the correlation analysis between the three orientations of compassion, as measured by the CEAS-PL, are presented in Table 5.

**Table 5:** Inter-correlations (Pearson’s r) between scales of the CEAS-PL.

	Study 1 (*N* = 593)				Study 2 (*N* = 238)				Study 3 (*N* = 378)			
Measures	*M*	*SD*	CtoO	CfO	*M*	*SD*	CtoO	CfO	*M*	*SD*	CtoO	CfO
**Compassion to Self**	40.31	10.10	.16^***^	.22^***^	40.78	11.31	.60^***^	.55^***^	37.31	10.63	.17^**^	.16^**^
**Compassion to Others**	71.82	11.64	–	.74^***^	65.43	15.01	–	.87^***^	63.55	15.26	–	.80^***^
**Compassion from Others**	64.78	11.29	–	–	60.14	13.52	–	–	57.51	12.99	–	–

**p < .01,

***p < .001

### Validity

To assess the convergent and discriminant validity of the CEAS-PL, we compared it with other measures of compassion and self-compassion. Additionally, we aimed to explore the relationships between the three CEAS-PL scales and various attributes of compassion such as empathic concern, perspective-taking, and emotion regulation. Furthermore, to examine the connections between the three compassion orientations and outcome variables that compassion may influence, we investigated the relationships between the CEAS-PL scales and depression, anxiety, and stress (expecting negative correlations), as well as satisfaction with life and well-being (anticipating positive correlations). We also examined the associations between the three scales of the CEAS-PL and dispositional traits such as personality traits and attachment orientation. We expected moderate correlations with certain traits (extraversion, emotional stability, close attachment orientation) and no significant correlations with others (e.g., conscientiousness).

The correlations between the CEAS-PL scales and all criterion variables are presented in Table 6.

**Table 6:** Pearson’s correlations between scales of the CEAS-PL and other variables.

	Sample 1 (*N* = 583)					Sample 2 (*N* = 238)				
Measures	*M*	*SD*	Compassion to Self	Compassion to Others	Compassion from Others	*M*	*SD*	Compassion to Self	Compassion to Others	Compassion from Others
Self-Compassion Scale										
**Self-Kindness**	3.03	.96	.64^***^	.01	.15^***^	3.01	.92	.41^***^	.25^***^	.31^***^
**Common Humanity**	3.02	.87	.45^***^	.02	.11^*^	3.18	.96	.35^***^	.25^***^	.28^***^
**Mindfulness**	3.09	.83	.57^***^	.05	.09^*^	3.16	.87	.41^***^	.25^***^	.28^***^
Compassion Scale										
**Kindness**	16.86	2.51	.05	.65^***^	.46^***^	15.98	3.17	.19^**^	.48^***^	.44^***^
**Common Humanity**	16.92	2.17	.14^***^	.26^***^	.18^***^	15.84	2.97	.24^***^	.34^***^	.32^***^
**Mindfulness**	17.90	1.91	.12^**^	.49^***^	.31^***^	16.34	2.87	.24^***^	.46^***^	.41^***^
**Indifference**	16.76	2.64	.04	.55^***^	.39^***^	15.38	3.27	.10	.20^***^	.19^**^
**Compassion (total)**	17.11	1.75	.11^**^	.66^***^	.45^***^	15.94	2.26	.29^***^	.54^***^	.50^***^
Empathic Sensitivity Scale										
**Empathic Concern**	43.16	6.12	-.04	.59^***^	.47^***^	39.51	5.86	.27^***^	.45^***^	.42^***^
**Personal Distress**	24.56	5.99	-.39^***^	.08	.02	24.50	5.43	-.26^***^	.07	.05
**Perspective Taking**	36.05	4.60	.14^***^	.45^***^	.35^***^	32.91	4.73	.34^***^	.52^***^	.49^***^
Revised Adult Attachment Scale										
**Close**	21.23	5.23	.15^***^	.23^***^	.32^***^	19.62	4.52	.32^***^	.28^***^	.28^***^
**Depend**	18.81	5.52	.29^***^	.13^**^	.45^***^	17.74	4.54	.16^*^	.09	.18^**^
**Anxiety**	16.69	6.79	-.32^***^	.05	-.14^***^	17.19	6.61	-.16^*^	.02	.04
Emotion Regulation Question.										
**Emotion Regulation**	40.56	9.12	.29^***^	.02	-.04	45.12	12.17	.41^***^	.28^***^	.32^***^
DAAS-21										
**Depression**	13.48	5.64	-.42^***^	.01	-.12^**^	14.71	6.27	-.16^*^	.13^*^	.10
**Anxiety**	12.50	5.38	-.30^***^	.11^**^	-.05	13.16	5.69	-.10	.09	.09
**Stress**	15.68	5.58	-.39^***^	.06	-.09^*^	15.15	5.60	-.15^*^	.12	.10
Satisfaction with Life Scale										
**Satisfaction with Life**	21.68	6.50	.43^***^	.03	.19^***^	19.18	6.37	.32^***^	.20^**^	.21^**^
Psychological Well-Being Scales										
**Autonomy**	11.84	2.24	.29^***^	.03	-.01	11.46	2.05	.29^***^	.18^**^	.12
**Environmental Mastery**	11.93	2.20	.42^***^	.04	.10^*^	11.65	2.00	.42^***^	.32^***^	.31^***^
**Personal Growth**	13.64	1.73	.30^***^	.14^***^	.14^***^	11.79	1.85	.28^***^	.32^***^	.25^***^
**Positive Relations With Others**	14.11	2.16	.19^***^	.34^***^	.45^***^	12.45	2.26	.27^***^	.23^***^	.23^***^
**Purpose in Life**	14.13	2.06	.15^***^	.12^**^	.15^***^	12.29	2.06	.24^***^	.17^**^	.09
**Self-Acceptance**	11.38	2.72	.51^***^	.05	.20^***^	10.43	2.43	.37^***^	.16^*^	.16^*^
Ten Item Personality Inventory										
**Extraversion**	10.49	2.85	.27^***^	.13^**^	.19^***^	9.75	3.24	.36^***^	.26^***^	.29^***^
**Agreeableness**	11.11	2.27	.17^***^	.26^***^	.25^***^	10.81	2.28	.16^*^	.23^***^	.22^***^
**Consciousness**	10.63	2.93	.27^***^	.04	.06	11.60	2.32	.30^***^	.25^***^	.25^***^
**Neuroticism**	8.79	3.29	-.50^***^	.09^*^	-.04	7.76	3.05	-.30^***^	-.16^*^	-.13^*^
**Openness to experience**	10.58	2.24	.18^***^	.16^***^	.09^*^	9.13	2.33	.09	.11	.07

*p < .05,

**p < .01,

***p < .001

## Discussion

The aim of this study was to evaluate the psychometric properties of the Polish version of the Compassionate Engagement and Action Scales (CEAS-PL). Our findings generally support the reliability and validity of the CEAS-PL across different samples, though some limitations were observed. These results are broadly consistent with previous studies on the original CEAS and its other language versions.

### Factor structure and reliability

The structure of the original version of the CEAS, developed by Gilbert et al. [18], aligns with the concept that compassion involves two categories of skills: motivated attention to suffering and efforts to alleviate and prevent suffering, which operate across three flows of compassion: showing compassion to others, receiving compassion from others, and self-compassion. Each of the three scales measuring these flows consists of two sections: six items assessing Compassionate Engagement and four items assessing Compassionate Action, which are integrated into a higher-order factor. This structure was confirmed in two samples, one from the United States and one from Portugal, during the questionnaire’s initial validation [18].

Subsequent studies, including validations of various language versions of the CEAS, have yielded differing factor analysis results. For the Turkish version [22], a study conducted on the UK general population [26], and the study by Murfield et al. [70] involving 171 English-speaking family carers of older adults, mostly residents of Australia, the three scales were confirmed with two distinct factor structures each. In contrast, for the Japanese version [21], a single-factor structure was confirmed for each of the three flows of compassion. Halamová et al. [24], using data from a relatively large Slovak sample (*N* = 638), tested the CEAS structure through CFA and the Exploratory Structural Equation Modeling (ESEM) framework, which integrates EFA within a structural equation modeling (SEM) framework [71]. For all three scales in the Slovak version, the bifactor ESEM model demonstrated the best fit.

The results of these studies are challenging to compare due to differences in methodology, as well as the fact that achieving good model fit in some studies required removing certain items. Nevertheless, the findings of our analyses are broadly consistent with those outlined above.

Across both Sample 1 (students, *N* = 593) and Sample 2 (general population, *N* = 238), the models for the Compassion to Others and Compassion from Others scales demonstrated overall good fit to the data, based on key indices. These models consistently showed the lowest χ²/df, RMSEA, and SRMR values, as well as the highest TLI and CFI values across both samples, confirming the robustness of the bifactor model structure for these constructs.

In contrast, the results for the Compassion to Self scale were less consistent between the two samples. In Sample 1, the bifactor model showed the best fit among the tested models but displayed higher RMSEA and χ²/df values compared to the other scales, indicating that while it fit the data relatively well, further refinement is needed to improve its fit. For Sample 2, the bifactor model failed to converge, likely due to insufficient sample size or model complexity. Notably, other tested models for the Compassion to Self scale in Sample 2, such as the two-factor and hierarchical models, demonstrated good fit indices, suggesting viable alternative structures for this scale.

Overall, the bifactor models for Compassion to Others and Compassion from Others demonstrated consistent and strong performance across both samples, underscoring their robustness. Meanwhile, the Compassion to Self scale presents areas for improvement, particularly regarding its bifactor structure.

The internal consistency of the CEAS-PL scales, measured by Cronbach’s α, was satisfactory, with values ranging from.80 to.94, except for the Engagement component of the Compassion for Self scale, which showed low reliability (.56 to.65). However, this scale in our version was shortened to only three items, and the low reliability coefficient may be attributed to the reduced length of the scale [40].

The Cronbach’s alpha values obtained for the CEAS-PL scales across the three samples were very similar and not lower than those reported by Asano et al. [21] for the Japanese version of the CEAS and Lindsey et al. [26] for the UK general population.

Gilbert et al. [18], as well as the authors of the Turkish [22] and Slovak [24] versions, provided Cronbach’s alpha values only for the Engagement and Action components rather than for the full scales. Comparing these component-level results with our findings reveals similar values overall, with larger differences observed for Compassion to Others—Engagement in the Slovak version (α = .65, compared to.80–.84 in our study) and Compassion to Self—Engagement (α = .90 in the Slovak version,.70 in the Turkish version, and.56–.65 in our study). However, comparisons for the Compassion to Self—Engagement component are challenging, as the Polish version includes only three items, whereas the other versions retain six.

The results of the test-retest reliability analysis indicate low correlation coefficients (.57 to.64) between the first and second measurements. However, given the long interval between measurements, these values may still be considered acceptable for constructs expected to show natural variability over time [40].

The results of the intraclass correlations between the CEAS-PL scales only partially support the expectation that the different orientations of compassion are somewhat distinct from one another. In Study 1 and Study 3, as anticipated, the correlations between Compassion to Self and the other scales were moderate. However, the correlation between Compassion to Others and Compassion from Others was high. In Study 2, all intraclass correlations were high. For comparison, the inter-correlations between the three CEAS scales in Kleissen’s [23] study ranged from.28 to.53, with the highest value observed for the correlation between Compassion to Others and Compassion from Others. In the Japanese version [21], the inter-correlations ranged from.21 to.37, while in Lindsey et al.’s [26] study, they ranged from.28 to.43. The findings from our studies suggest that the use of the CEAS-PL may encounter issues with multicollinearity, particularly due to the high correlations observed between Compassion to Others and Compassion from Others. The findings from our studies suggest that the use of the CEAS-PL may be affected by issues of multicollinearity, particularly due to the high correlations observed between the Compassion to Others and Compassion from Others scales. One potential explanation for this high correlation is that participants may have interpreted compassion given to others and compassion received from others as two closely related facets of the same interpersonal orientation toward compassion. Additionally, cultural factors specific to the Polish population may have contributed to the perception of these two forms of compassion as inherently interconnected—possibly due to stronger social norms of reciprocity or heightened expectations of mutuality in interpersonal relationships [72]. Consequently, future research may benefit from revising or clarifying the wording of questionnaire items to more effectively distinguish between these two dimensions of compassion.

### Construct validity

Correlation analyses performed across three different samples revealed results consistent with expectations. These findings also highlight distinct relationships for the various orientations of compassion, as measured by the CEAS-PL.

The Compassion for Self scale showed strong and moderate positive correlations with positively oriented subscales from Neff’s Self-Compassion Scale [14]. However, there are low or no correlations between Compassion to Others and Compassion from Others with the self-compassion subscales. In contrast, the Compassion to Others and Compassion from Others scales showed low positive correlations with self-compassion and strong or moderate positive correlations with experiencing kindness, a sense of common humanity, mindfulness, and reduced indifference toward others’ suffering measured by the Compassion Scale [15]. These results confirm the expected similarities between the CEAS-PL and other tools measuring analogous types of compassion.

The relationships between the CEAS-PL scales and the components of the Empathic Sensitivity Scale are also interesting. Empathic concern and perspective taking correlate relatively highly and positively with Compassion to Others and Compassion from Others, but not with Compassion to Self, confirming that the other-oriented scales of the CEAS predict affective and cognitive empathy towards others. Compassion to Self, however, negatively correlates with personal distress, which, according to Davis’s multidimensional theory of empathy [49], is associated with a tendency to avoid experiencing discomfort in the face of another person’s suffering.

The individual flows of compassion measured by the CEAS-PL are weakly related to attachment orientations, with these relationships being negative for anxiety orientation, and positive for close orientation, as well as (for most CEAS-PL scales) for depend orientation.

The ability to regulate emotions, defined as possessing strategies that individuals use to enhance, sustain, or diminish emotional reactions [54], is positively but moderately related to the level of Compassion to Self (both samples) and other CEAS-PL scales (Sample 2). This is consistent with the expectation that the ability to manage one’s own emotions is a fundamental aspect of compassion. However, the ability to regulate emotions alone does not mean that a person will have a high level of compassion, as other important factors, such as motivation to engage in collaborative, helping behaviours, also play a crucial role [11].

The CEAS-PL scales are moderately positively correlated with life satisfaction and well-being, with differences observed in the strength of these relationships for the three flows of compassion and components of well-being. For example, Self-Acceptance is relatively most strongly associated with the level of Compassion to Self and weakly or not at all with the other flows of compassion. In contrast, Positive Relations with Others are similarly (moderate positive correlation) related to each of the CEAS-PL scales, but (only for Sample 1) most strongly with the other-oriented CEAS-PL scales. The CEAS-PL results are also negatively related to indicators of negative emotions measured by the DASS21. As in the Japanese sample [21], these correlations were moderate or weak, and relatively strongest for Compassion to Self.

The relationships between the CEAS-PL results and personality traits were, as expected, moderate or weak. All three scales were moderately positively correlated with extraversion and agreeableness, and negatively with neuroticism. In Sample 1, weak or moderate positive correlations between all flows of compassion and openness to experiences were also observed. However, the lack of correlation between CEAS-PL scales and openness to experiences in Sample 2 is somewhat surprising. It seems that individual differences in compassion can only be moderately explained by differences arising from partially genetically determined dispositional personality traits. Compassion can be deepened and developed throughout a person’s life through participation in workshops, therapy, and self-development (10,11,71). Our results appear consistent with these assumptions.

### Strengths, Limitations and Future Research Directions

One of the strengths of the studies used to analyze the psychometric properties of the Polish version of the CEAS was the inclusion of three different samples: one consisting of students and two drawn from the general population. The study procedure involved the administration of a relatively large number of questionnaires, which enabled a comprehensive analysis of the scale’s convergent and discriminant validity.

However, as with any study, this research has certain limitations that should be acknowledged. The data were collected using a cross-sectional design and self-report questionnaires, which may be subject to biases such as social desirability or inaccurate self-assessment. Furthermore, data collection was conducted online, without full standardization of conditions. Although control procedures, such as attention-check questions, were implemented to minimize errors, it cannot be entirely ruled out that some participants may have responded inattentively to the questionnaire items. Additionally, while the samples were relatively large, they may still have been insufficient for conducting confirmatory factor analyses (CFA) with the desired level of statistical power. It should also be noted that there was a predominance of women in the study groups, accounting for 73.3% of the participants, which may limit the generalizability of the findings to more gender-balanced or male-dominated populations.

The fit of the structural models for each of the CEAS-PL scales was established after the removal of four items: three from the Compassion to Self scale and one from the Compassion from Others scale. Similar situations have been reported in previous studies.

The removal of the item “I am emotionally moved by my distressed feelings or situations” (CfS4) has also been recommended by Asano et al. [21] and Lindsey et al. [26]. Additionally, Ari et al. [22] reported that this item exhibited very low factor loadings in their study. Similarly, Asano et al. [21], like in our study, removed the item CfS2 “I notice, and am sensitive to my distressed feelings when they arise in me.” Lindsey et al. [26] also excluded CfS5 “I tolerate the various feelings that are part of my distress” and CtO5 “I tolerate the various feelings that are part of other people’s distress”, both of which were removed in our analysis as well. Furthermore, Asano et al. [21] and Ari et al. [22] suggested the removal of CfS8, while Lindsey et al. [26] proposed removing CfO5 “Others tolerate my various feelings that are part of my distress.”.

Concerns about each of the items removed in our study have also been identified in at least one other language version of the CEAS. This pattern suggests that the challenges with these items may not be limited to issues of cultural adaptation but could instead reflect broader structural difficulties in how these aspects of compassion are conceptualized and operationalized.

Murfield et al. [70] and Lindsey [26] suggest further exploratory work on items related to tolerating upsetting emotional states. Each of the three CEAS subscales includes one item addressing this aspect, two of which (CfS5 and CtO5) were also removed from the model in our study, further highlighting this issue. Moreover, challenges in measuring tolerance of distress have been noted in other compassion scale studies [73], indicating a broader concern in the conceptualization and operationalization of tolerating various feelings as an aspect of compassion.

Notably, the item “I am emotionally moved by my distressed feelings or situations” was removed during CFA in four different samples—UK, Turkey, Japan, and Poland—to improve model fit. We believe this item also warrants further attention in future work aimed at refining the CEAS. Qualitative studies, in particular, could provide valuable insights into how individuals interpret the content of this item. It is possible that the phrase “emotionally moved by” is not understood as an element of self-compassion but rather as an indicator of heightened negative emotions and stress—an interpretation that may not align with the intended construct of self- compassion.

The concerns described above regarding the potential misfit of certain CEAS items, observed across studies conducted in various countries and using different language versions, underscore the need for a reconceptualization of the scales measuring Compassion for Others, Compassion from Others, and especially Compassion for Self.

It is important to note that using scales with removed items in research can make it more challenging to compare findings across different versions of the tool. In the case of the Polish version, this issue is particularly relevant for the Compassion for Self – Engagement subscale, from which three out of six items were removed. Researchers should take into account the potential issue of incomplete comparability between the Polish version and other versions of the CEAS.

An additional limitation of the Polish version of the CEAS is the higher-than-expected correlation between the scores for Compassion for Others and Compassion from Others, which does not align with theoretical assumptions and exceeds the levels reported in other studies. Future research could address this by conducting qualitative studies to investigate how participants interpret items measuring these constructs, potentially uncovering cultural or linguistic factors contributing to their overlap. Furthermore, a reassessment of the Polish version’s cultural adaptation, with a focus on refining or rewording items that may overlap conceptually, could help clarify distinctions between the two constructs.

### Implications

Besides the aforementioned psychometric properties, it is also of note that the CEAS-PL may be particularly useful for practitioners (i.e., psychotherapists, clinical psychologists, and other helping professionals) to determine how patients cope in each of the dimensions (flows) of compassion. This may in turn influence choosing adequate therapeutic interventions., and may be a valuable insight, alongside the Fears of Compassion Scales [74] for the purpose of clinical formulations [75], and as one of the tools in monitoring the therapeutic process, effectiveness and efficacy at its various stages, i.e., outcome and progress monitoring [76,77].

Considering the psychometric limitations of the Polish version of the Compassion for Self scale, we recommend exercising caution when using this scale in research and diagnostic contexts, as it may carry a potential risk of errors. Similar concerns were raised by Halamová et al. [24] regarding the Slovak version of the CEAS. We believe that further refinement and reconstruction of this scale are needed to enhance its reliability and validity.

The CEAS is a versatile tool that allows for the assessment of overall scores or specific components of each scale, depending on the purpose of its use. According to Gilbert et. al [18], the scales “can be used as single factor scales or, for more detailed explorations, as separate sub-scales (engagement and actions) for each orientation”. In Compassion Focused Therapy (CFT), engagement reflects connecting with feelings of compassion, while action involves taking compassionate steps. These processes are interconnected but distinct, and CFT interventions address three flows of compassion—self-compassion, compassion for others, and openness to receiving compassion—each trainable independently to meet individual needs (5,10). To evaluate the effectiveness of CFT, selecting the CEAS subscale relevant to the targeted flow can provide focused insights. Additionally, analyzing changes in engagement or action within a specific flow may offer a more detailed understanding of therapeutic outcomes.

## Conclusion

The findings of this study provide valuable insights into the psychometric properties of the Polish version of the Compassionate Engagement and Action Scales (CEAS-PL). Despite some limitations, such as inconsistencies in the Compassion for Self scale and slightly higher correlations between Compassion to Others and Compassion from Others in the others studies, the CEAS-PL demonstrates satisfactory reliability and validity across diverse samples. These results align with previous studies on the original CEAS and its language adaptations, underscoring the utility of this tool in both research and clinical practice.

However, given the identified limitations, we recommend using the CEAS-PL with caution, particularly in studies aiming to draw detailed conclusions from the Compassion for Self scale. Further refinement of this scale appears necessary, especially to enhance its psychometric robustness and cross-cultural applicability. Several factors may contribute to these difficulties. For example, processing information about how one feels toward others or how others feel about oneself is very different from processing internal states. Items that ask people to tune into personal distress may trigger avoidance rather than compassion engagement. Indeed, Gilbert et al. [18] found that ‘sensitivity and being emotionally moved by distress, without the other aspects of compassion, are positively associated with a range of mood and negative self-evaluative variables’ [17]. As we have concluded, this poses a challenge for compassion, because, clearly, sensitivity, attention to, and awareness of suffering are core processes of compassion. However, as has been found in mindfulness research [78], if individuals are only attentive and sensitive to the suffering (observant), they risk ruminating and dwelling on pain which increases distress, rather than reducing it. Hence, being compassionate elevates sensitivity, which needs to be contextualised by qualities such as tolerance, ability to reflect on one’s distress with understanding, being non-judgmental, and being motivated to work with one’s distress. These findings lead to questions about the use of self-reports to measure different states of mind. That different constituent parts can exhibit different patterns does not mean that those parts should be removed from the scale. For example, while one can remove items of sensitivity for factor analysis fit, this does not necessarily indicate that it is clinically justified.

The CEAS-PL shows potential as a practical instrument for assessing the three flows of compassion and guiding therapeutic interventions. Future efforts to refine the Compassion for Self scale and to explore cultural and linguistic factors in item interpretation will enhance its applicability and accuracy. Overall, this study highlights the CEAS-PL’s promise as a valuable tool for understanding and fostering compassion in varied contexts.

## Supporting information

S1 Fig Three-factor model (Model II) with excluded items. (TIF)

S2 Fig The alternative measurement models for the Compassion for Self scale. (TIF)

S3 Fig The alternative measurement models for the Compassion from Others scale. (TIF)

S1 File Compassionate Engagement and Action Scales – Polish version. (PDF)

S2 File CEAS – original and Polish item wordings with item codes used in the paper. (PDF)

S1 Dataset SPSS Data File for Sample 1. (SAV)

S2 Dataset SPSS Data File for Sample 2. (SAV)

S3 Dataset SPSS Data File for Sample 3. (SAV)
