# Supplementary figures and images for: The development of the Polish version of the Compassionate Engagement and Action Scales
Source: PLoS One. 2025 May 15;20(5):e0323687. doi: 10.1371/journal.pone.0323687 (PMC12080769; doi:10.1371/journal.pone.0323687)

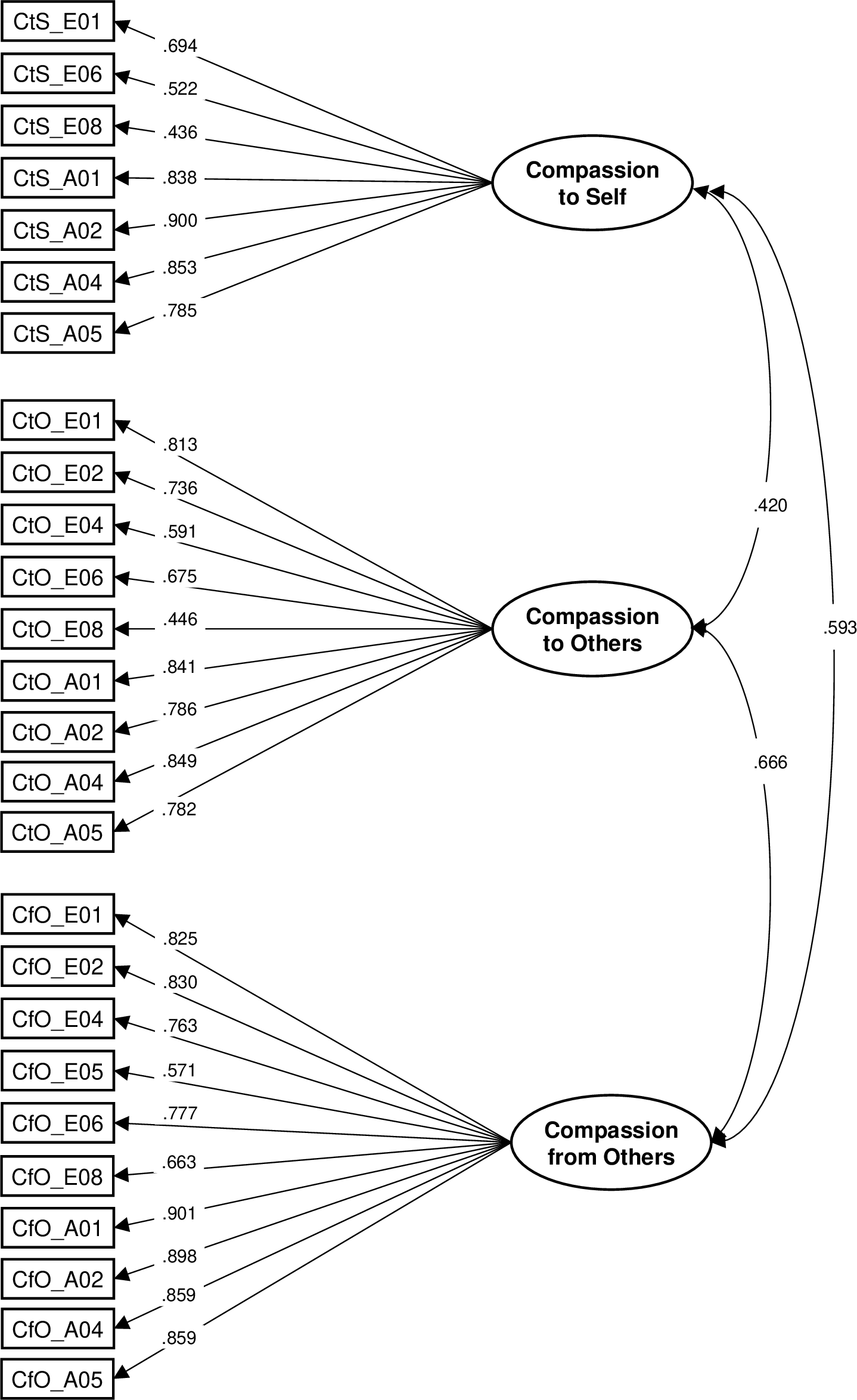

Supplement: S1 Fig — (TIF) [file pone.0323687.s001.tif]

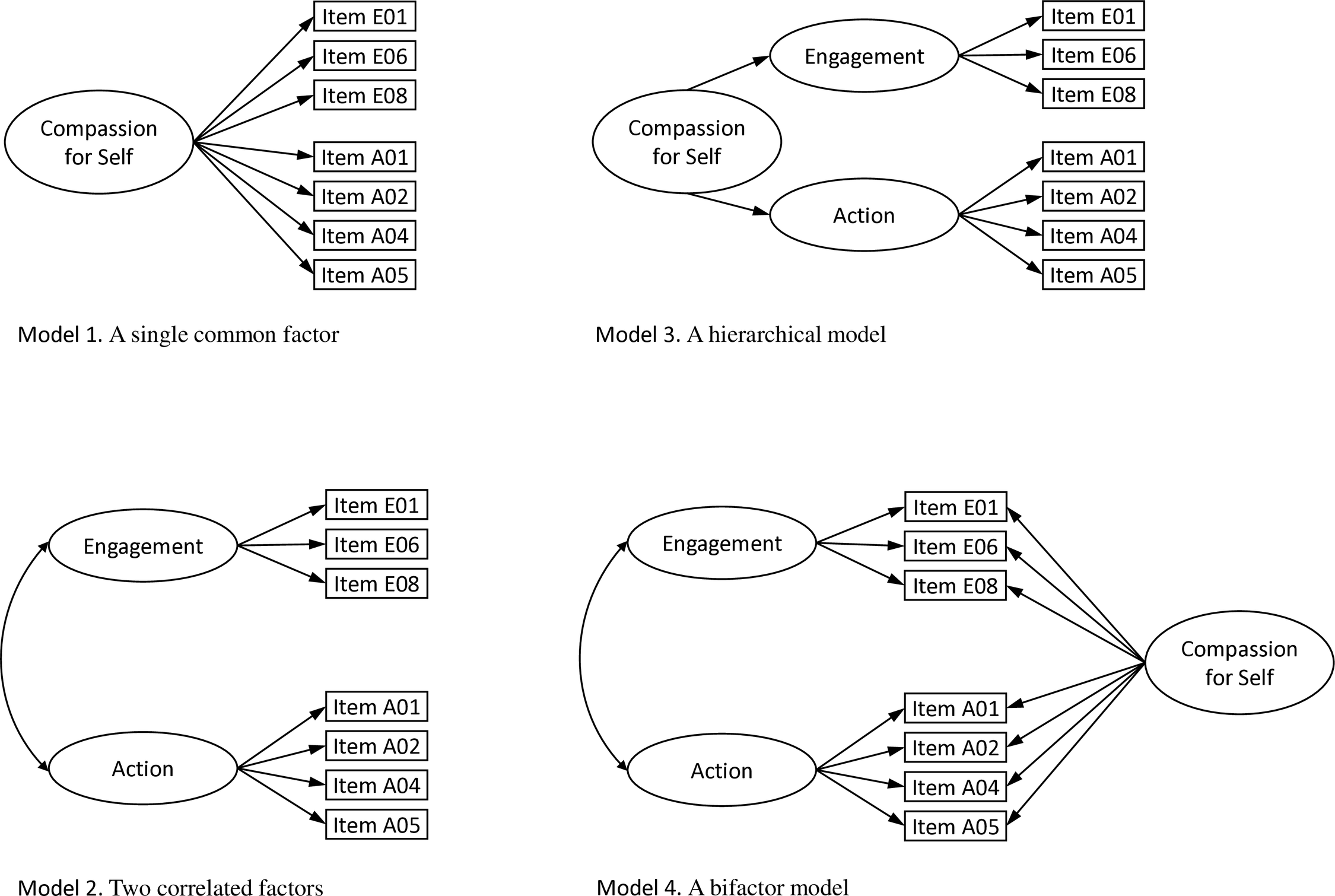

Supplement: S2 Fig — (TIF) [file pone.0323687.s002.tif]

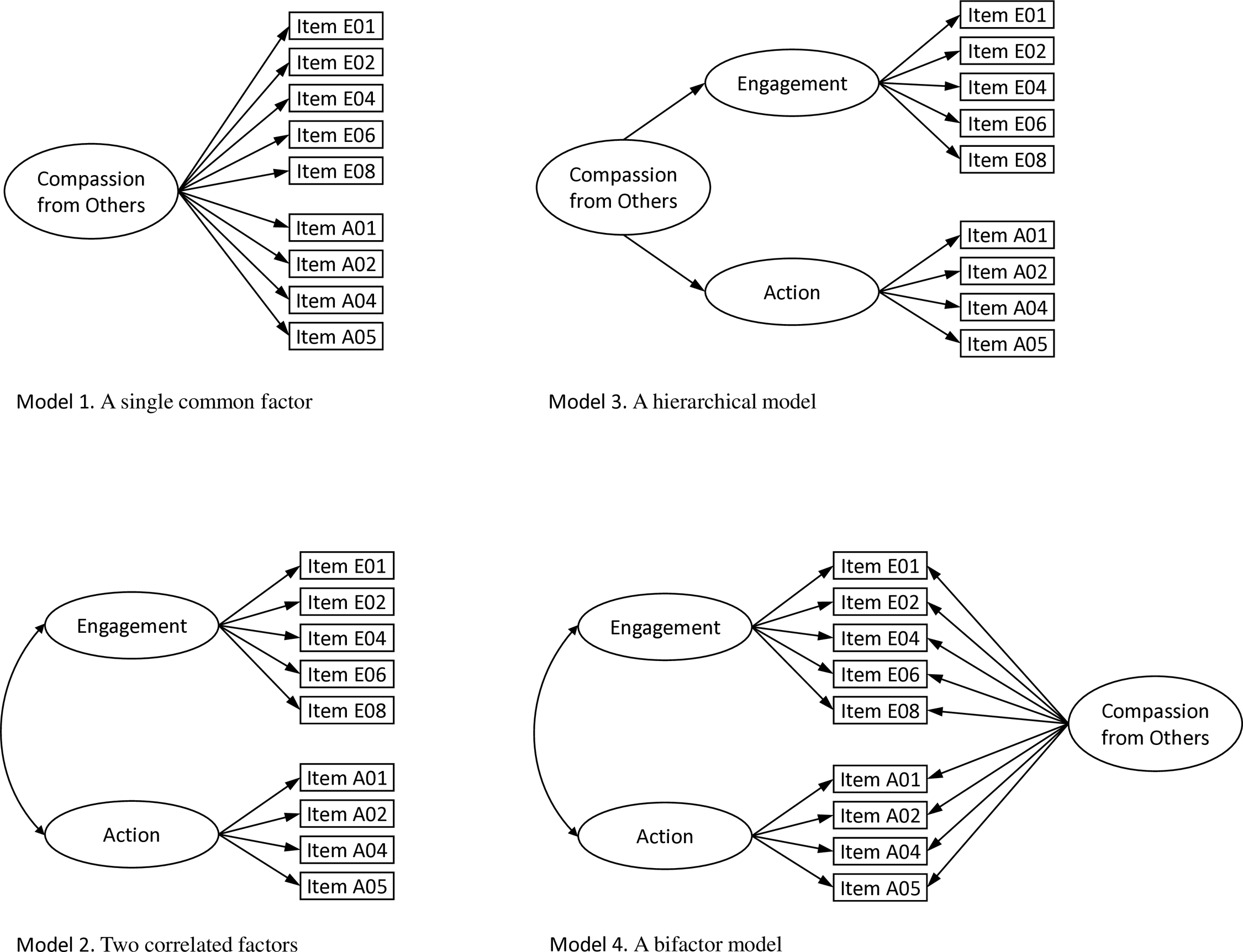

Supplement: S3 Fig — (TIF) [file pone.0323687.s003.tif]
