# Supplementary material for: The development of the Polish version of the Compassionate Engagement and Action Scales
Source: PLoS One. 2025 May 15;20(5):e0323687. doi: 10.1371/journal.pone.0323687 (PMC12080769; doi:10.1371/journal.pone.0323687)
Supplement: S1 File — (PDF) [file pone.0323687.s004.pdf]

Skale Współczującego Zaangażowania i Działania (CEAS-PL)

Compassionate Engagement and Action Scales: Gilbert i in., 2017. Polska adaptacja: Zięba i in., 2025

Współczucie wobec siebie

Kiedy doświadczamy trudności, niepowodzeń, rozczarowań lub strat, będących źródłem silnego napięcia emocjonalnego i dyskomfortu (dystresu), możemy sobie z nimi radzić na różne sposoby. Jesteśmy zainteresowani w jakim stopniu ludzie potrafią być **współczujący wobec samych siebie**. Współczucie definiujemy jako „wrażliwość na cierpienie swoje lub innych wraz z zaangażowaniem w próby jego złagodzenia lub zapobiegnięcia." Oznacza to, że istnieją dwa aspekty współczucia. *Pierwszy* z nich jest zdolnością utrzymywania motywacji do zajmowania się rzeczami lub uczuciami, które są trudne, w przeciwieństwie do prób uniknięcia ich lub stłumienia. *Drugim* aspektem współczucia jest zdolność skupienia się na tym co jest dla nas pomocne. Tak samo jak lekarz ze swoim pacjentem. Zadaniem pierwszego jest bycie zmotywowanym i zdolnym do skupienia uwagi na bólu oraz zrozumienie go (lub znalezienie sposobu jak to zrobić). Zadaniem drugiego jest bycie zdolnym do podjęcia działań które będą pomocne. Poniżej znajduje się lista pytań, które dotyczą tych dwóch aspektów współczucia. Przeczytaj uważnie każde zdanie i pomyśl w jaki sposób dotyczy ono Pana / Pani w sytuacji przeżywania silnego napięcia emocjonalnego. Proszę, aby ocenił /-a Pan / Pani poniższe twierdzenia używając następującej skali:

Nigdy 1 2 3 4 5 6 7 8 9 10 **Zawsze**

Sekcja 1 – Poniższe pytania odnoszą się do Pana / Pani motywacji i zdolności do zaangażowania się w radzenie sobie z napięciem i dyskomfortem (dystresem), kiedy ich doświadczasz. A zatem:

Kiedy jestem w stanie dystresu (napięcia i dyskomfortu)…

1. Jestem *zaangażowany* w zmniejszanie i zapobieganie mojego dystresu (napięcia i dyskomfortu) kiedy się pojawia.
Nigdy 1 2 3 4 5 6 7 8 9 10 **Zawsze**

2. *Zauważam* i jestem *wrażliwy/a* na moje uczucia napięcia i dyskomfortu gdy się pojawiają.
Nigdy 1 2 3 4 5 6 7 8 9 10 **Zawsze**

3. *Unikam* myślenia o moim dystresie (napięciu i dyskomforcie), staram się odwrócić od nich uwagę oraz wyrzucić je z głowy.
Nigdy 1 2 3 4 5 6 7 8 9 10 **Zawsze**

4. *Czuję się poruszony emocjonalnie* w stresujących sytuacjach.
Nigdy 1 2 3 4 5 6 7 8 9 10 **Zawsze**

5. *Toleruję* różnorodne uczucia, które składają się na moje napięcie i dyskomfort (dystres).
Nigdy 1 2 3 4 5 6 7 8 9 10 **Zawsze**

6. *Zastanawiam* się nad moimi uczuciami napięcia i dyskomfortu i *rozumiem* je.
Nigdy 1 2 3 4 5 6 7 8 9 10 **Zawsze**

7. *Nie jestem w stanie znieść* napięcia i dyskomfortu.
Nigdy 1 2 3 4 5 6 7 8 9 10 **Zawsze**

8. *Akceptuję, nie krytykuję i nie oceniam* mojego napięcia i dyskomfortu.
Nigdy 1 2 3 4 5 6 7 8 9 10 **Zawsze**

Sekcja 2 – Te pytania odnoszą się do tego w jaki sposób aktywnie wykorzystujesz współczucie w radzeniu sobie z emocjami, myślami i sytuacjami, które są źródłem Twojego dystresu (napięcia i dyskomfortu). A zatem:

Kiedy jestem w stanie dystresu (napięcia i dyskomfortu)…

1. Kieruję moją *uwagę* na to co może być dla mnie pomocne.
Nigdy 1 2 3 4 5 6 7 8 9 10 **Zawsze**

2. *Myślę* o pomocnych sposobach na radzenie sobie z moim napięciem i dyskomfortem i je znajduję
Nigdy 1 2 3 4 5 6 7 8 9 10 **Zawsze**

3. Nie wiem jak sobie pomóc.
Nigdy 1 2 3 4 5 6 7 8 9 10 **Zawsze**

4. Podejmuje działania i robię rzeczy, które będą dla mnie pomocne.
Nigdy 1 2 3 4 5 6 7 8 9 10 **Zawsze**

5. Tworzę dla siebie wewnętrzne *wsparcie, pomoc i otuchę*.
Nigdy 1 2 3 4 5 6 7 8 9 10 **Zawsze**

Współczucie wobec Innych

Kiedy inni ludzie doświadczają trudności, niepowodzeń, rozczarowań lub strat, będących źródłem silnego napięcia i dyskomfortu (dystresu), możemy sobie radzić z ich udręką na różne sposoby. Jesteśmy zainteresowani w jakim stopniu ludzie potrafią być **współczujący dla innych**. Współczucie definiujemy jako „wrażliwość na cierpienie swoje lub innych wraz z zaangażowaniem w próby jego złagodzenia lub zapobiegnięcia." Oznacza to, że istnieją dwa aspekty współczucia. *Pierwszy* z nich jest zdolnością utrzymywania motywacji do zajmowania się rzeczami lub uczuciami, które są trudne, w przeciwieństwie do prób uniknięcia ich lub stłumienia. *Drugim* aspektem współczucia jest zdolność skupienia się na tym co jest dla nas pomocne. Tak samo jak lekarz ze swoim pacjentem. Zadaniem pierwszego jest bycie zmotywowanym i zdolnym do skupienia uwagi na bólu oraz zrozumienie go (lub znalezienie sposobu jak to zrobić). Zadaniem drugiego jest bycie zdolnym do podjęcia działań które będą pomocne. Poniżej znajduje się lista pytań, które dotyczą tych dwóch aspektów współczucia. Dlatego też proszę o przeczytanie uważnie każdego zdanie i pomyślenie w jaki sposób dotyczy ono Pana / Pani w sytuacji kiedy **ludzie w Pana / Pani życiu** odczuwają silne napięcie emocjonalne. Proszę, aby ocenił /-a Pan / Pani poniższe twierdzenia używając następującej skali:

Nigdy 1 2 3 4 5 6 7 8 9 10 **Zawsze**

Sekcja 1 – Poniższe pytania odnoszą się do Pana / Pani motywacji i zdolności do zaangażowania się w radzenie sobie z napięciem i dyskomfortem (dystresem), kiedy doświadczają ich inni ludzie. A zatem:

Kiedy inni ludzie są w stanie dystresu (napięcia i dyskomfortu)…

1. Jestem *zaangażowany* w zmniejszanie i zapobieganie dystresu (napięcia i dyskomfortu) u innych ludzi, kiedy się pojawia.
Nigdy 1 2 3 4 5 6 7 8 9 10 **Zawsze**

2. *Zauważam* i jestem wrażliwy/a na uczucia napięcia i dyskomfortu u innych ludzi, gdy się pojawiają.
Nigdy 1 2 3 4 5 6 7 8 9 10 **Zawsze**

3. *Unikam* myślenia o napięciu i dyskomforcie u innych ludzi, staram się odwrócić od niego uwagę oraz wyrzucić je z głowy.
Nigdy 1 2 3 4 5 6 7 8 9 10 **Zawsze**

4. Jestem *poruszony/a* przez napięcie i dyskomfort u innych ludzi lub wywołujące je sytuacje.
Nigdy 1 2 3 4 5 6 7 8 9 10 **Zawsze**

5. *Znoszę* różnorodne uczucia, które składają się na napięcie i dyskomfort (dystres) u innych ludzi.
Nigdy 1 2 3 4 5 6 7 8 9 10 **Zawsze**

6. *Zastanawiam* się nad uczuciami napięcia i dyskomfortu u innych ludzi i *rozumiem* je.
Nigdy 1 2 3 4 5 6 7 8 9 10 **Zawsze**

7 Nie jestem w stanie znieść napięcia i dyskomfortu u innych ludzi.
Nigdy 1 2 3 4 5 6 7 8 9 10 **Zawsze**

8. *Akceptuję, nie krytykuję i nie oceniam* napięcia i dyskomfortu u innych ludzi.
Nigdy 1 2 3 4 5 6 7 8 9 10 **Zawsze**

Sekcja 2 – Te pytania odnoszą się do tego w jaki sposób aktywnie wykorzystujesz współczucie gdy inni ludzie doświadczają dystresu (napięcia i dyskomfortu). A zatem:

Kiedy inni ludzie są w stanie dystresu (napięcia i dyskomfortu)…

1. Kieruję moją *uwagę* na to co może być pomocne dla innych.
Nigdy 1 2 3 4 5 6 7 8 9 10 **Zawsze**

2. *Myślę* o pomocnych sposobach na radzenie sobie z ich napięciem i dyskomfortem i je *znajduję*.
Nigdy 1 2 3 4 5 6 7 8 9 10 **Zawsze**

3. Nie wiem jak pomóc innym ludziom gdy doświadczają napięcia i dyskomfortu.
Nigdy 1 2 3 4 5 6 7 8 9 10 **Zawsze**

4. Podejmuje działania i robię rzeczy, które będą pomocne dla innych.
Nigdy 1 2 3 4 5 6 7 8 9 10 **Zawsze**

5. Wyrażam uczucia *wsparcia, uczynności i otuchy* wobec innych ludzi..
Nigdy 1 2 3 4 5 6 7 8 9 10 **Zawsze**

Współczucie od innych

Kiedy doświadczamy trudności, niepowodzeń, rozczarowań lub strat, będących źródłem silnego napięcia emocjonalnego, inni ludzie mogą sobie radzić z naszą udręką na różne sposoby. Jesteśmy zainteresowani w jakim stopniu czuje Pan / Pani, że **ważne osoby w Pana / Pani życiu mogą być współczujące wobec Pana / Pani napięcia i dyskomfortu (dystresu)**. Współczucie definiujemy jako „wrażliwość na cierpienie swoje lub innych wraz z zaangażowaniem w próby jego złagodzenia lub zapobiegnięcia." Oznacza to, że istnieją dwa aspekty współczucia. *Pierwszy* z nich jest zdolnością utrzymywania motywacji do zajmowania się rzeczami lub uczuciami, które są trudne, w przeciwieństwie do prób uniknięcia ich lub stłumienia. *Drugim* aspektem współczucia jest zdolność skupienia się na tym co jest dla nas pomocne. Tak samo jak lekarz ze swoim pacjentem. Zadaniem pierwszego jest bycie zmotywowanym i zdolnym do skupienia uwagi na bólu oraz zrozumienie go (lub znalezienie sposobu jak to zrobić). Zadaniem drugiego jest bycie zdolnym do podjęcia działań które będą pomocne. Poniżej znajduje się lista pytań, które dotyczą tych dwóch aspektów współczucia. Dlatego też prosimy o przeczytanie uważnie każdego zdania i pomyślenie w jaki sposób dotyczy ono **ważnych osób w Pana / Pani życiu** w sytuacjach kiedy doświadczasz silnego napięcia emocjonalnego. Proszę, aby ocenił /-a Pan / Pani poniższe twierdzenia używając następującej skali:

Nigdy 1 2 3 4 5 6 7 8 9 10 **Zawsze**

Sekcja 1 – Poniższe pytania dotyczą tego jak bardzo Pana / Pani zdaniem inni ludzie są zaangażowani w Pana / Pani uczucia napięcia i dyskomfortu (dystresu) gdy ich Pan / Pani doświadcza. A zatem:

Kiedy jestem w stanie dystresu (napięcia i dyskomfortu)…

1. Inni ludzie są *zaangażowani* w zmniejszanie i zapobieganie mojego dystresu (napięcia i dyskomfortu) kiedy się pojawia.
Nigdy 1 2 3 4 5 6 7 8 9 10 **Zawsze**

2. Inni ludzie *zauważają* i są *wrażliwi* na moje uczucia napięcia i dyskomfortu gdy się pojawiają.
Nigdy 1 2 3 4 5 6 7 8 9 10 **Zawsze**

3 Inni ludzie *unikają* myślenia o moim dystresie (napięciu i dyskomforcie), starają się odwrócić od niego uwagę oraz wyrzucić je z głowy.
Nigdy 1 2 3 4 5 6 7 8 9 10 **Zawsze**

4. Inni ludzie są *poruszeni* przez moje napięcie i dyskomfort lub wywołujące je sytuacje.
Nigdy 1 2 3 4 5 6 7 8 9 10 **Zawsze**

5. Inni ludzie *znoszą* różnorodne uczucia, które składają się na moje napięcie i dyskomfort (dystres).
Nigdy 1 2 3 4 5 6 7 8 9 10 **Zawsze**

6. Inni ludzie *zastanawiają* się nad moimi uczuciami napięcia i dyskomfortu i *rozumieją* je.
Nigdy 1 2 3 4 5 6 7 8 9 10 **Zawsze**

7. Inni ludzie nie są w stanie znieść mojego napięcia i dyskomfortu.
Nigdy 1 2 3 4 5 6 7 8 9 10 **Zawsze**

8. Inni ludzie *akceptują, nie krytykują i nie oceniają* mojego napięcia i dyskomfortu.
Nigdy 1 2 3 4 5 6 7 8 9 10 **Zawsze**

Sekcja 2 – Te pytania odnoszą się do tego w jaki sposób inni ludzie aktywnie wykorzystują współczucie w radzeniu sobie z emocjami, myślami i sytuacjami, które są źródłem Pana / Pani dystresu (napięcia i dyskomfortu). A zatem:

Kiedy jestem w stanie dystresu (napięcia i dyskomfortu)…

1. Inni ludzie kierują swoją *uwagę* na to co może być dla mnie pomocne.

Nigdy 1 2 3 4 5 6 7 8 9 10 **Zawsze**

2. Inni ludzie *myślą* o sposobach na poradzenie sobie przeze mnie z napięciem i dyskomfortem.

Nigdy 1 2 3 4 5 6 7 8 9 10 **Zawsze**

3. Inni ludzie nie wiedzą jak mi pomóc kiedy doświadczam napięcia i dyskomfortu.

Nigdy 1 2 3 4 5 6 7 8 9 10 **Zawsze**

4. Inni ludzie podejmują *działania* i robią rzeczy, które będą dla mnie pomocne.

Nigdy 1 2 3 4 5 6 7 8 9 10 **Zawsze**

5. Inni ludzie okazują mi *wsparcie, uczynność i otuchę.*

Nigdy 1 2 3 4 5 6 7 8 9 10 **Zawsze**
