# Supplementary material for: The development of the Polish version of the Compassionate Engagement and Action Scales
Source: PLoS One. 2025 May 15;20(5):e0323687. doi: 10.1371/journal.pone.0323687 (PMC12080769; doi:10.1371/journal.pone.0323687)
Supplement: S2 File — (PDF) [file pone.0323687.s005.pdf]

Item code	Self-Compassion Scale	
	Section 1: Self-Compassion – Engagement	
CtoS_E01	I am motivated to engage and work with my distress when it arises.	Jestem zaangażowany w zmniejszanie i zapobieganie mojego dystresu (napięcia i dyskomfortu) kiedy się pojawia.
CtoS_E02	I notice, and am sensitive to my distressed feelings when they arise in me.	Zauważam i jestem wrażliwy/a na moje uczucia napięcia i dyskomfortu gdy się pojawiają.
CtoS_E03	I avoid thinking about my distress and try to distract myself and put it out of my mind.	Unikam myślenia o moim dystresie (napięciu i dyskomforcie), staram się odwrócić od nich uwagę oraz wyrzucić je z głowy.
CtoS_E04	I am emotionally moved by my distressed feelings or situations.	Czuję się poruszony emocjonalnie w stresujących sytuacjach.
CtoS_E05	I tolerate the various feelings that are part of my distress.	Toleruję różnorodne uczucia, które składają się na moje napięcie i dyskomfort (dystres).
CtoS_E06	I reflect on and make sense of my feelings of distress.	Zastanawiam się nad moimi uczuciami napięcia i dyskomfortu i rozumiem je.
CtoS_E07	I do not tolerate being distressed.	Nie jestem w stanie znieść napięcia i dyskomfortu.
CtoS_E08	I am accepting, non-critical and non-judgemental of my feelings of distress.	Akceptuję, nie krytykuję i nie oceniam mojego napięcia i dyskomfortu.
Section 2: Self-Compassion – Action		
CtoS_A01	I direct my attention to what is likely to be helpful to me.	Kieruję moją uwagę na to co może być dla mnie pomocne.
CtoS_A02	I think about and come up with helpful ways to cope with my distress.	Myślę o pomocnych sposobach na radzenie sobie z moim napięciem i dyskomfortem i je znajduję
CtoS_A03	I don't know how to help myself.	Nie wiem jak sobie pomóc.
CtoS_A04	I take the actions and do the things that will be helpful to me.	Podejmuje działania i robię rzeczy, które będą dla mnie pomocne.
CtoS_A05	I create inner feelings of support, helpfulness and encouragement.	Tworzę dla siebie wewnętrzne wsparcie, pomoc i otuchę.
Compassion to Others Scale		
Section 1: Compassion to Others – Engagement		
CtoO_E01	I am motivated to engage and work with other peoples' distress when it arises.	Jestem zaangażowany w zmniejszanie i zapobieganie dystresu (napięcia i dyskomfortu) u innych ludzi, kiedy się pojawia.
CtoO_E02	I notice and am sensitive to distress in others when it arises.	Zauważam i jestem wrażliwy/a na uczucia napięcia i dyskomfortu u innych ludzi, gdy się pojawiają.
CtoO_E03	I avoid thinking about other peoples' distress, try to distract myself and put it out of my mind.	Unikam myślenia o napięciu i dyskomforcie u innych ludzi, staram się odwrócić od niego uwagę oraz wyrzucić je z głowy.
CtoO_E04	I am emotionally moved by expressions of distress in others.	Jestem poruszony/a przez napięcie i dyskomfort u innych ludzi lub wywołujące je sytuacje.
CtoO_E05	I tolerate the various feelings that are part of other people's distress.	Znoszę różnorodne uczucia, które składają się na napięcie i dyskomfort (dystres) u innych ludzi.
CtoO_E06	I reflect on and make sense of other people's distress.	Zastanawiam się nad uczuciami napięcia i dyskomfortu u innych ludzi i rozumiem je.
CtoO_E07	7 I do not tolerate other peoples' distress.	7 Nie jestem w stanie znieść napięcia i dyskomfortu u innych ludzi.
CtoO_E08	I am accepting, non-critical and non-judgemental of others people's distress.	Akceptuję, nie krytykuję i nie oceniam napięcia i dyskomfortu u innych ludzi.

Section 2: Compassion to Others – Action		
CtoO_A01	I direct attention to what is likely to be helpful to others.	Kieruję moją uwagę na to co może być pomocne dla innych.
CtoO_A02	I think about and come up with helpful ways for them to cope with their distress.	Myślę o pomocnych sposobach na radzenie sobie z ich napięciem i dyskomfortem i je znajduję.
CtoO_A03	I don't know how to help other people when they are distressed.	Nie wiem jak pomóc innym ludziom gdy doświadczają napięcia i dyskomfortu.
CtoO_A04	I take the actions and do the things that will be helpful to others.	Podejmuje działania i robię rzeczy, które będą pomocne dla innych.
CtoO_A05	I express feelings of support, helpfulness and encouragement to others.	Wyrażam uczucia wsparcia, uczynności i otuchy wobec innych ludzi..
Compassion from Others Scale		
Section 1: Compassion from Others – Engagement		
CfO_E01	Other people are actively motivated to engage and work with my distress when it arises.	Inni ludzie są zaangażowani w zmniejszanie i zapobieganie mojego dystresu (napięcia i dyskomfortu) kiedy się pojawia.
CfO_E02	Others notice and are sensitive to my distressed feelings when they arise in me.	Inni ludzie zauważają i są wrażliwi na moje uczucia napięcia i dyskomfortu gdy się pojawiają.
CfO_E03	3 Others avoid thinking about my distress, try to distract themselves and put it out of	3 Inni ludzie unikają myślenia o moim dystresie (napięciu i dyskomforcie), starają się odwrócić od niego uwagę oraz wyrzucić je z głowy.
CfO_E04	Others are emotionally moved by my distressed feelings.	Inni ludzie są poruszeni przez moje napięcie i dyskomfort lub wywołujące je sytuacje.
CfO_E05	Others tolerate my various feelings that are part of my distress.	Inni ludzie znoszą różnorodne uczucia, które składają się na moje napięcie i dyskomfort (dystres).
CfO_E06	Others reflect on and make sense of my feelings of distress.	Inni ludzie zastanawiają się nad moimi uczuciami napięcia i dyskomfortu i rozumieją je.
CfO_E07	Others do not tolerate my distress.	Inni ludzie nie są w stanie znieść mojego napięcia i dyskomfortu.
CfO_E08	Others are accepting, non-critical and non-judgemental of my feelings of distress.	Inni ludzie akceptują, nie krytykują i nie oceniają mojego napięcia i dyskomfortu.
Section 2: Compassion from Others – Action		
CfO_A01	Others direct their attention to what is likely to be helpful to me.	Inni ludzie kierują swoją uwagę na to co może być dla mnie pomocne.
CfO_A02	Others think about and come up with helpful ways for me to cope with my distress.	Inni ludzie myślą o sposobach na poradzenie sobie przeze mnie z napięciem i dyskomfortem.
CfO_A03	Others don't know how to help me when I am distressed	Inni ludzie nie wiedzą jak mi pomóc kiedy doświadczam napięcia i dyskomfortu.
CfO_A04	Others take the actions and do the things that will be helpful to me.	Inni ludzie podejmują działania i robią rzeczy, które będą dla mnie pomocne.
CfO_A05	Others treat me with feelings of support, helpfulness and encouragement.	Inni ludzie okazują mi wsparcie, uczynność i otuchę.
